# On the Diarrhœa of Enteric or Typhoid Fever

**Published:** 1867-09

**Authors:** George Johnson

**Affiliations:** Physician to King’s College Hospital


### On the Diarrhœa of Enteric or Typhoid Fever.

By Dr. George Johnson. Physician to King's College Hospital.

[During a quarter of a century Dr. Johnson has seen two different and opposite plans of treating the diarrhœa of fever. The late Dr. Todd gave repeated doses of opiates and powerful astringents. When the stools were frequent it was a common practice to give an enema of starch with laudanum, twice, thrice, or evon oftener, in the course of the day. This practice was not attended with satisfactory results.]

All this has been much changed during the last few years. There has been no change in the type of typhoid fever; the disease is, in every respect, the same as in former years. There is the same intestinal ulceration; but the intestinal symptoms are far less troublesome. There is much less of obstinate diarrhœa, much less of distressing tympanitis; and this amelioration of symptoms is coincident with a complete change of practice. I have described the former mode of treatment. Our practice now is, as a rule, to leave the diarrhœa alone, and rarely to give opiates or other astringents to check it. You will understand, of course, that I am speaking only of the practice in my own wards. It is a most unquestionable fact that, since the discontinuance of the opiate and astringent treatment, the diarrhœa and the other intestinal symptoms have been far less troublesome to the attendants, and far less distressing to the patients.

And it appears to me that the explanation of these different results is not difficult. In most cases of typhoid fever, there must be more or less of diarrhœa; for there is ulceration of the bowel, and, as a consequence of this, morbid secretions are poured out, which irritate the bowel and have to be expelled. This is obvious, without entering upon any theoretical considerations. If now, while this morbid process is going on in the intestines, repeated opiates are given, either by the mouth or by the rectum, the effect is certainly not to stop or to check the ulcerative process in the bowel, nor to prevent the pouring out of morbid secretions from the ulcerated surfaces; but to lessen the sensibility of the bowel, and so to retain the morbid secretions until they decompose, give off offensive gases, and thus become a fresh source of irritation and distress. I attribute the unfavorable results of this practice mainly to the effect of the opiates in preventing or retarding the expulsion of the offensive secretions from the bowel.

Not long since some of you had the opportunity of seeing the effect of discontinuing the astringent treatment in the case of a young woman who was admitted at about the end of the second week of typhoid fever. She had been under the care of a friend and former pupil of my own, and he told us that she had been treated by logwood and laudanum every six hours, yet, in spite of this, the diarrhœa had been profuse and frequent up to the very time of her admission into Twining Ward. I directed her to be put upon the usual fever diet, and to have a dose of colored water three times a day. The troublesome diarrhœa ceased immediately; the bowels acted only once or twice a-day. She made a good recovery; and my friend frankly admitted that the "let-alone plan" had been much more successful than his opiate and astringent treatment.

In our endeavor to explain the undoubted fact, that the intestinal symptoms of typhoid fever are now much less troublesome than in past times, it is right to mention that in some other particulars our treatment has been modified. We now give much less medicine of every kind than we formerly did; and, in particular, we avoid the risk of irritating the bowels by repeated doses of mineral acids. We give alcoholic stimulants more sparingly and with more discrimination. As a rule, we give none during the early stages of the fever; when I am convinced that their indiscreet employment often increases febrile excitement, cerebral oppression, and gastro-intestinal irritation. In short, our chief reliance now in the treatment of this fever is upon rest in bed, with good nursing, judicious feeding, and stimulants when necessary. Our patients are fed mainly upon milk, beef-tea, eggs, and arrow-root.

If you refer to Trousseau's *Clinique Medicale*, (tome i. p. 258,) you will find that his practice, when the stools of typhoid fever are frequent and abundant, is to give saline purgatives—either sulphate of soda or a Seidleitz powder. This treatment he thinks especially indicated, when the diarrhœa is associated with much flatulent distention of the bowels, and in such cases he repeats the dose several times. If, after this, the diarrhœa continue, he gives what he calls absorbent powders—nitrate of bismuth in combination with chalk, and in some cases small doses of nitrate of silver, but he makes no mention of opium as a remedy in this class of cases.

When the intestines become much distended by a mixture of air and liquid, the relief which follows evacuation of the bowels is often great and permanent. This may sometimes be effected by a laxative enema, but in most cases more surely by a tablespoonful of castor oil combined with a few drops of laudanum in some aromatic water. In such cases, if we can get rid of irritating secretions by a mild evacuant, we are acting on the principle which should continually guide us in the treatment of typhoid fever, namely: to ensure as much as possible of rest for the diseased intestine. The intestines in these cases may be irritated by uncalled-for drugs, by injudicious feeding, by the untimely or excessive administration of alcoholic stimulants, by the accumulation of morbid secretions within the bowels, by muscular exertion on the part of the patient, or by rough pressure over the abdomen on the part of the practitioner. All these known sources of irritation and of injury ought, therefore, to be most carefully avoided.

In conclusion, let me say emphatically that, when peritonitis is threatened, or actually present, whether the result of perforation of the bowel or of the ulcerative process extending deeply into the tissues, our main reliance is upon absolute rest, light hot fomentations over the abdomen, and opium in full and frequent doses. I have seen cases apparently the most desperate recover under this plan of treatment; one case in particular, that of a girl, eleven years of age, in whom all the symptoms of perforation of the bowel were present. In such a case, when recovery takes place, however sudden and severe may have been the onset of the peritoneal symptoms, it must of course remain doubtful whether perforation of the bowel had actually occurred.—*British Medical Journal.*

